# Restoring tibial obliquity for kinematic alignment in total knee arthroplasty: conventional versus patient-specific instrumentation

**DOI:** 10.1007/s00402-023-04845-7

**Published:** 2023-03-20

**Authors:** Maria Anna Smolle, Amir Koutp, Clemens Clar, Lukas Leitner, Andreas Leithner, Patrick Sadoghi

**Affiliations:** Department of Orthopaedics and Trauma, Medical University of Graz, Graz, Austria

**Keywords:** Total knee arthroplasty, Kinematic alignment, Patient-specific instrumentation, Conventional instrumentation, Tibial obliquity

## Abstract

**Introduction:**

In total knee arthroplasty (TKA), tibial obliquity-restoration using kinematic alignment (KA) poses a major difference to conventional mechanical alignment. This study aimed at analysing the accuracy of conventional instrumentation (CI) versus patient-specific instrumentation (PSI) to restore anatomic tibial obliquity measured by the medial proximal tibial angle (MPTA) on conventional X-rays.

**Materials and Methods:**

One-hundred patients were randomized to receive CI (*n* = 50) or PSI (*n* = 50) for TKA. Further 100 patients received CI without randomisation, resulting in 200 patients in total (127 women, mean age: 70.7 (range: 48–90 years). Pre- and postoperative X-rays were measured twice by two observers with a 2-week break in-between. Inter- and intraclass correlations were calculated and postoperative tibial obliquity compared to preoperative anatomy.

**Results:**

In 150 patients with CI, no case with tibial obliquity-deviation greater than 2° was found, whilst 21.3% (*n* = 32) and 0.7% (*n* = 1) of cases and had a deviation of 0°–1°, and 1°–2°, respectively. In the remaining 78.0% (*n* = 117), tibial obliquity was restored. In 50 patients with PSI, no single case with a deviation greater than 1° was found. Sixty percent (*n* = 30) had a deviation of 0°–1°. In the remaining 40.0% (*n* = 20), no deviation from preoperative measurements was found. Consequently, CI resulted in a significantly smaller change in tibial obliquity from preoperative to postoperative than PSI (*p* < 0.001). Inter- and intra-class correlations showed a substantial agreement (any ICC > 0.90).

**Conclusion:**

Both conventional and patient-specific instrumentation revealed adequate results with respect to restoring tibial obliquity in kinematically aligned TKA, with conventional instrumentation achieving superior results.

## Introduction

For decades, surgeons tried to achieve neutral tibial alignment with a medial proximal tibial angle (MPTA) of 90° and, therefore, were not trained to align the tibia according to this physiological MTPA equivalent to a mean of 2.9° with a range from 20.5° of valgus to 20.5° of varus [17]. The theoretical advantage of neutral mechanical alignment—with the mechanical axis passing mid-line—is the even load distribution in the medial and lateral femorotibial compartment with consecutively reduced risk for implant loosening and wear [3–5, 13, 16]. In literature, however, this is discussed controversially [1]. In addition, mechanical alignment does not necessarily depict patients’ individual knee anatomy. In fact, only 0.1% of TKA patients actually have neutral femoral and tibial mechanical axes, whilst the vast majority presents with some degree of variation [2, 8]. Even more, according to a study by *Bellemans *et al*.* a constitutional varus knee, defined as natural mechanical alignment of at least 3° varus, can be found in 17% and 32% of asymptomatic adult women and men, respectively [3]. Upon TKA, neutral mechanical alignment in these patients would overcorrect the pre-existing varus, and eventually require some sort of medial soft tissue release [3]. Based on these observations, the philosophy of kinematic alignment (KA) was first proposed by *Howell *et al*.* aiming at a more patient-tailored reconstruction of the mechanical axis [8, 10]. First long-term results on KA are encouraging, with implant survivorship of 97.4% at 10 years [11].

Methods to achieve KA in TKA include caliper verification (as proposed by *Howell *et al*.*), robotics, computer-aided surgery, or patient-specific instrumentation (PSI) [8, 15]. In comparison to conventional instrumentation with standard blocks, PSI uses customised cutting blocks based on 3D models deriving from preoperative magnetic resonance imaging (MRI) or computed tomography (CT) scans [14], to achieve a more individualized reconstruction of preoperative knee anatomy [17]. However, the definite decision which instrumentation should be used to achieve KA still has to be made.

The aim of the current study was, therefore, to evaluate the accuracy of conventional instrumentation (CI) versus patient-specific instrumentation (PSI) with respect to restoration of tibial obliquity (based on mechanical medial proximal tibial angle [MPTA]) measured on conventional X-rays. It was hypothesized that PSI would be superior to conventional instrumentation with respect to accuracy of restoring tibial obliquity.

## Material and methods

**Fig. 1:**
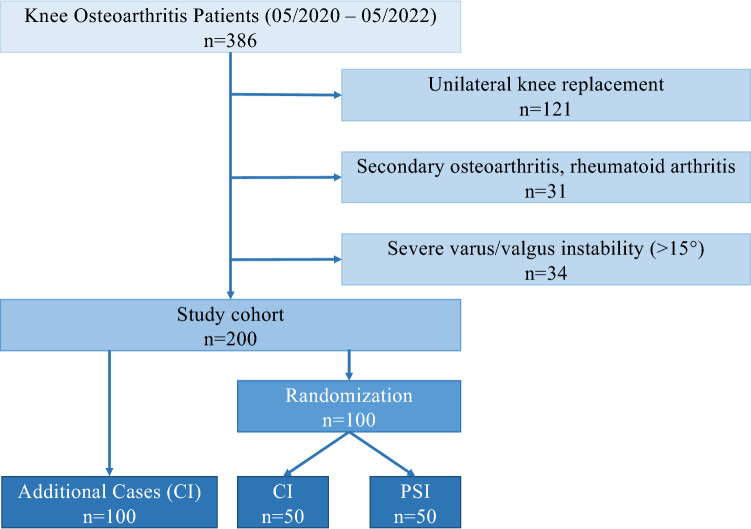
Flowchart of patient selection with in- and exclusion criteria at the respective time points

This study was approved by the institutional review board (*blinded for review*). Of 386 patients undergoing TKA at a single high-volume orthopaedic surgery centre due to end-stage osteoarthritis (grade IV according to Kellgren and Lawrence) of 2 out of 3 compartments of their joint, one-hundred patients were prospectively included between May 2020 and May 2022 and randomized into CI (*n* = 50) or PSI (*n* = 50) groups. Further 100 patients from the initial cohort directly underwent TKA with CI (Fig. 1). Mean age at surgery of the entire cohort (*n* = 200) was 70.7 (range: 48–90) years, and 127 were women (63.5%). All procedures were performed by one experienced senior knee surgeon.

### Inclusion and exclusion criteria

All patients with primary knee osteoarthritis above the age of 50 years requiring TKA were potentially eligible. Patients younger than 50 years of age, those with a history of tibial or femoral fracture, osteotomy, septic arthritis, rheumatoid arthritis, or previous (partial) knee arthroplasty at the side of osteoarthritis, were excluded (Fig. 1). Prior to study participation, all patients gave their written informed consent.

### Surgical technique

**Fig. 2:**
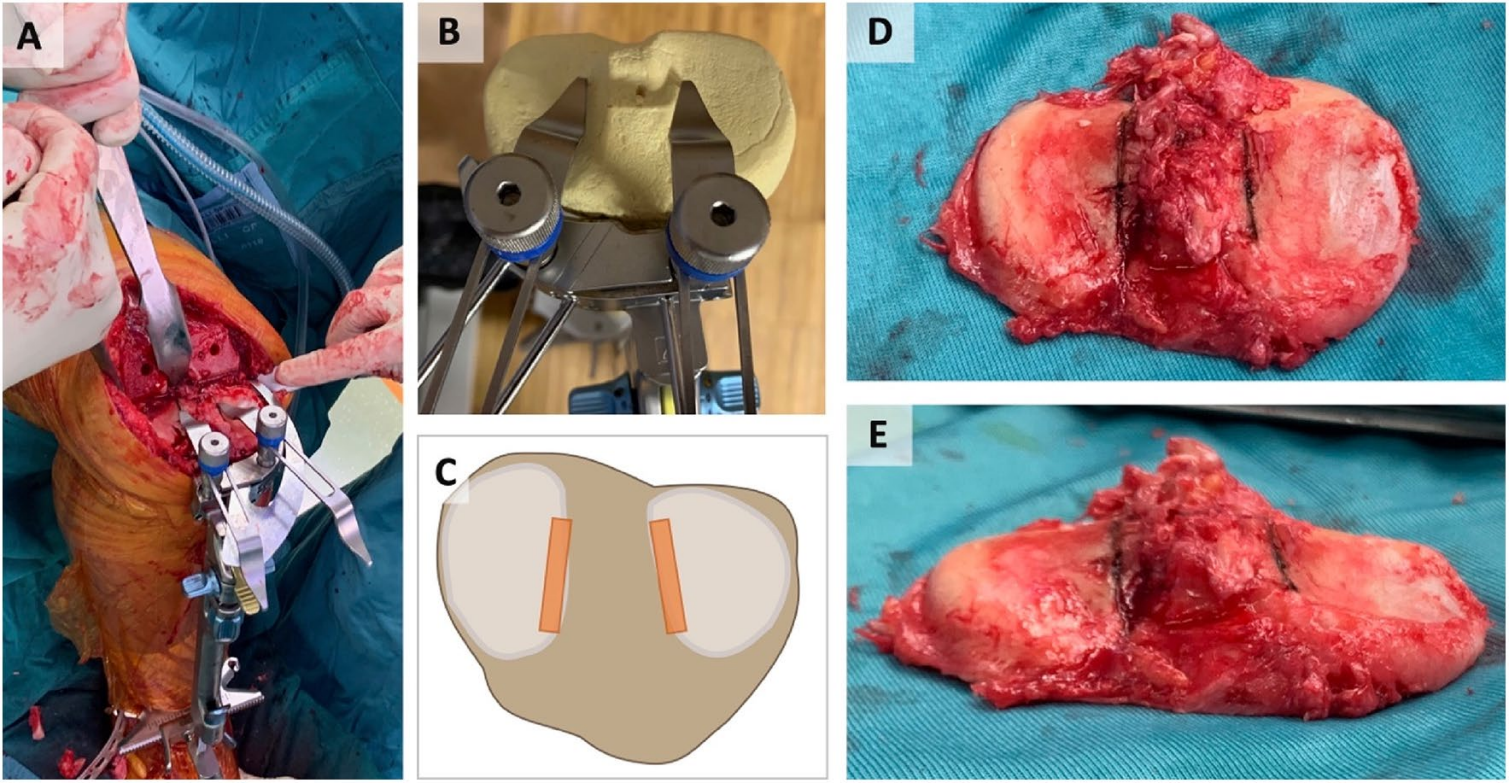
Illustration of restoring tibial obliquity in kinematic alignment with the calipered technique presented by *Howell* [14] using 2 tibial stylus of 8 mm medial and lateral to the tibial spine on the border without cartilage wear to restore pre-arthritic anatomy of the joint. **A** Extramedullary instrumentation with 2 stylus is attached to the tibia in situ. **B** Illustration of extramedullary instrumentation with 2 stylus on a sawbone. **C** Graphic of the region of interest medial and lateral of the tibial spine illustrated with blue lines. (**D**/**E**) Resected tibial plateau illustrating obliquity and native tibial slope

All patients were operated using the medial pivot cemented GMK Sphere system (*Medacta, Castel San Pietro, Switzerland*). The group with CI was operated on according to the calipered technique by Howell [9]. Tibial obliquity was measured preoperatively with respect to the MPTA on X-ray. After finishing the femur with the calipered technique [9], the tibial obliquity was established using two tibial stylus with 8 mm to reference medial and lateral to the tibial spine at the border where no cartilage wear was evident to restore pre-arthritic anatomy (Fig. 2 A–E).

**Fig. 3:**
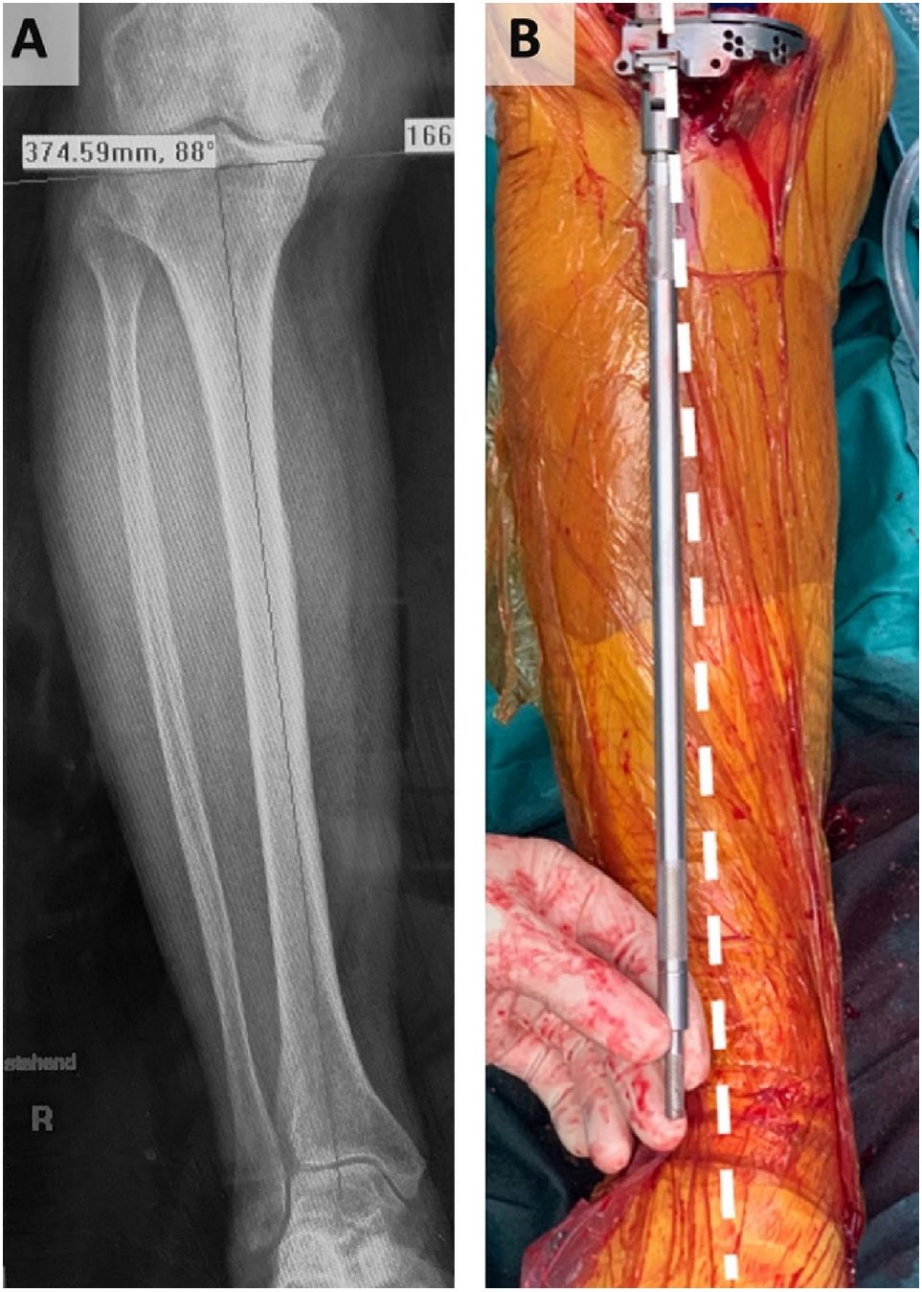
A.p. x-ray (**A**) of the tibia with measured tibial obliquity using the medial proximal tibial angle (2° of tibial varus) and intraoperative reevaluation (**B**) using a conventional alignment rod

Intraoperatively, tibial obliquity was checked for plausibility using a tibial alignment rod in line with preoperative X-ray of the tibia (Fig. 3). Tibial recuts were performed in case of inadequate extension or flexion gaps [9].

The group with PSI was operated according to the surgical technique provided by the company (*Medacta, Castel San Pietro, Switzerland*) using CT-based individual cutting jigs as presented by previous study groups [12]. Preoperative planning for the PSI group was performed by the company and double checked for verification by the senior surgeon in every single case. After finishing the distal femoral cut, the tibial cut was performed to evaluate the extension gap. Next, the femur was finished using the anatomic rotation as illustrated by the PSI guide.

For all patients in both techniques, the desired balance was equally balanced in full extension and laterally lax in 90 degrees of flexion according to the rationale of kinematic alignment [9–11].

### Radiological measurements

Tibial obliquity, defined as the MPTA, was measured by two independent investigators on pre- and postoperative x-rays of the entire limb using mediCAD 2D^®^ (*Hectec GmbH, Germany).* MPTA was defined as the medial angle formed between the tangent to the tibial plateau line and the tibial mechanical axis. Every investigator measured images twice in a random fashion. The second round of measurements took place at least 2 weeks after the first one. Drop-out rate of this study was 0%.

### Statistical analysis

For continuous variables with parametric and non-parametric distribution, means (with standard deviations [SDs]) and medians (with interquartile ranges [IQR]) were calculated. Definite values of pre- and postoperative tibial obliquity were calculated as means of the two mean measurements obtained by the two investigators. T-tests were used to assess differences in continuous variables between groups. To assess changes in continuous variables for more than two groups, Kruskal–Wallis tests were used. Changes in tibial obliquity (based on the MPTA) from pre- to postoperative were grouped into I. restoration of tibial obliquity, II. change 0°–1°, III. change 1°–2°, and IV. change > 2°.

Differences in tibial obliquity depending on treatment group were compared with chi-squared tests. Intraclass correlation coefficients (ICCs) between the reviewers were calculated for the two measurements. A-priori sample size calculation with a *p*-value of < 0.05 and a power greater than 80% revealed *n* = 50 cases per group as sufficient to detect a clinically relevant difference of 10% of deviation in the range from 0 to 1°, or above. A *p*-value of < 0.05 was considered statistically significant.

## Results

There were no significant differences in terms of gender distribution (*p* = 0.932), age at surgery (*p* = 0.290) or BMI (*p* = 0.141) between the two treatment groups (Table 1).

**Table 1:** Characteristics of the study population split by type of surgery

		Entire cohort (*n* = 200)	Conventional instrumentation (*n* = 150)	PSI (*n* = 50)	*P* value
Gender	*Male*	73 (36.5)	55 (36.7)	18 (36.0)	0.932
	*Female*	127 (63.5)	95 (63.3)	32 (64.0)	
Age at surgery (in years, mean ± SD)		70.7 ± 9.6	70.3 ± 9.5	71.9 ± 9.8	0.290
BMI (mean ± SD)		30.0 ± 5.3	30.3 ± 5.5	29.1 ± 4.6	0.141

Mean preoperative tibial obliquity amounted to 89.9° (range: 86°–93°). There was a significant difference in preoperative tibial obliquity depending on treatment group, with greater varus tibial obliquity in the CI (mean: 89.6° [range. 86°–93°]) than PSI group (mean: 90.6° [range: 88°–92°]; *p* < 0.001).

For the entire cohort, the postoperative tibial obliquity amounted to 89.9° (range: 85°–93°). There was a significant difference in postoperative tibial obliquity between treatment groups, again with greater varus obliquity in the CI (mean: 89.7° [range: 85°–93°]) than PSI group (mean: 90.5° [range: 88°–92°]; *p* < 0.001).

Overall, no change in tibial obliquity from pre- to postoperative larger than 2° was found. One patient presented with change in tibial obliquity between 1° and 2° (0.5%), and 62 with a change between 0° and 1° (31.0%). In the remaining 137 patients, preoperative tibial obliquity had been restored, with no deviation from preoperative measurement (68.5%).

Notably, in a significantly higher proportion of patients with CI, preoperative tibial obliquity had been restored (78.0%; *n* = 117) as compared to PSI (40.0%; *n* = 20; *p* < 0.001). Furthermore, 21.3% (*n* = 32) and 60.0% (*n* = 30) of patients with CI and PSI, respectively, presented with a tibial obliquity change between 0° and 1°. One change in tibial obliquity between 1° and 2° was observed with the conventional technique (0.7%). In detail, PSI rather resulted in valgus deviation from preoperative tibial obliquity than CI, i.e., a mean increase in tibial obliquity of 0.1° (range: − 1°–0.5°) was observed for PSI, compared to a mean decrease of − 0.1° (range: − 1.5°–1°) for CI. Moreover, change in tibial obliquity was comparable between men and women (*p* = 0.642), and did not alter significantly with increasing age (*p* = 0.169), or BMI (*p* = 0.320).

ICCs between reviewers for pre- (ICC = 0.988) and postoperative measurements (ICC = 0.999) showed excellent agreement.

## Discussion

According to the present study, both PSI and conventional instrumentation achieved adequate results with respect to restoring tibial obliquity in kinematically aligned TKA, and that CI was superior to PSI.

Till now, no study comparing KA with PSI and CI regarding change in tibial obliquity has been published in literature. However, some studies have focused on change in tibial obliquity comparing PSI-based kinematic with conventional mechanical alignment [6, 7, 18]. Radiological results of the present study (no change in tibial obliquity > 2°) are, for example, better than the ones observed by *Waterson *et al. in a in a prospective randomized controlled trial comparing KA with PSI (*n* = 71) vs. conventional mechanical alignment (*n* = 71) using the *Stryker Triathlon* (*Stryker Navigation, Kalamazoo, Michigan, US*) system [18]. They reported on 78% and 77% of kinematically and mechanically aligned TKAs to be within 3° of preoperative alignment, but did not provide statistical tests [18]. Other than in the present study, *Waterson *et al*.* referred to alignment measurements based on preoperative MRI scans that had been performed in the PSI group [18], whereas radiological alignment measurements of the present study were based on x-rays.

*Calliess *et al*.* performed a similar prospective randomized controlled trial in 100 patients with PSI and conventional mechanical instrumentation [6]. Comparable to our observations, they reported on larger deviations in postoperative limb alignment following PSI than conventional mechanical instrumentation, although not providing statistical tests [6].

*Dossett *et al*.* likewise carried out a prospective randomized controlled trial comparing KA with PSI (*n* = 44) and conventional mechanical alignment (*n* = 44) using the *Vanguard* (*Biomet Inc., Warsaw, Indiana, US*) system [7]. In their study, knee and limb alignment was comparable between groups, whilst knee joint alignment of patients in the kinematic group was significantly more valgus (by 1.9°), the tibial component significantly more varus (by 2.1°), and the femoral component significantly more valgus (by 2.2°) compared to the mechanically aligned group [7]. This corroborates our findings, with PSI leading to tibial obliquity in more valgus than CI, although we used CI with kinematic rather than mechanical alignment.

A limitation of the current study is the lack of clinical outcome data (e.g., WOMAC, KSS) to correlate with radiographic findings made. Furthermore, 3 patients had to be excluded secondarily due to missing postoperative knee images. However, the resulting drop-out rate of 0.5% may be considered negligible. Also, tibial obliquity was measured on a.p. x-rays only, whereas the sagittal alignment (i.e., tibial slope) had not been considered in the current study.

Furthermore, 50 patients were randomized into both groups. This process was further randomized due to visiting surgeons requesting a specific technique. In addition, 100 patients were operated without randomization on using the CI instrumentation due to lack of available CT scans for PSI.

## Conclusions

Both conventional and patient-specific instrumentation revealed adequate results with respect to restoring tibial obliquity in kinematically aligned TKA, with conventional instrumentation presenting superior results. In the future, the conventional technique may be preferred to PSI due to fewer radiation exposure and reduced costs. Nevertheless, further studies on larger cohorts might reveal the effect on potential outlier limitation using PSI.

## Data Availability

The datasets generated during and/or analysed during the current study are available from the corresponding author on reasonable request.
